# Investigation of Volatiles in Cork Samples Using Chromatographic Data and the Superposing Significant Interaction Rules (SSIR) Chemometric Tool

**DOI:** 10.3390/biom10060896

**Published:** 2020-06-11

**Authors:** Emili Besalú, Chantal Prat, Enriqueta Anticó

**Affiliations:** 1 Institute of Computational Chemistry and Catalysis, University of Girona, 17003 Girona, Spain; 2 Francisco Oller S.A., 17244 Cassà de la Selva, Spain; cprat@ollerfco.com; 3 Department of Chemistry, University of Girona, 17003 Girona, Spain

**Keywords:** cork, volatiles, GC-MS, SPME, superposing significant interaction rules

## Abstract

This study describes a new chemometric tool for the identification of relevant volatile compounds in cork by untargeted headspace solid phase microextraction and gas chromatography mass spectrometry (HS-SPME/GC-MS) analysis. The production process in cork industries commonly includes a washing procedure based on water and temperature cycles in order to reduce off-flavors and decrease the amount of trichloroanisole (TCA) in cork samples. The treatment has been demonstrated to be effective for the designed purpose, but chemical changes in the volatile fraction of the cork sample are produced, which need to be further investigated through the chemometric examination of data obtained from the headspace. Ordinary principal component analysis (PCA) based on the numerical description provided by the chromatographic area of several target compounds was inconclusive. This led us to consider a new tool, which is presented here for the first time for an application in the chromatographic field. The superposing significant interaction rules (SSIR) method is a variable selector which directly analyses the raw internal data coming from the spectrophotometer software and, combined with PCA and discriminant analysis, has been able to separate a group of 56 cork samples into two groups: treated and non-treated. This procedure revealed the presence of two compounds, furfural and 5-methylfurfural, which are increased in the case of treated samples. These compounds explain the sweet notes found in the sensory evaluation of the treated corks. The model that is obtained is robust; the overall sensitivity and specificity are 96% and 100%, respectively. Furthermore, a leave-one-out cross-validation calculation revealed that all of the samples can be correctly classified one at a time if three or more PCA descriptors are considered.

## 1. Introduction

The unique properties of cork, including long-lasting flexibility, hydrophobicity and gas permeability, make it still today the first choice for wine producers as a closure for bottled wine [1,2]. The complex chemical composition of cork, together with its importance in the wine industry, has resulted in the investigation of extractable compounds that can be released from the cork into the solution having become a subject of great interest [3,4,5]. Within the extractable components, volatiles including terpenes, alcohols, aldehydes and pyrazines, among others, deserve special attention [6,7,8,9,10]. The origin of these compounds is diverse, although they are normally ascribed to the transformation of lignin components, fatty acid oxidation or microbial degradation of fatty acids and aliphatic aldehydes [11,12]. The composition of the volatile fraction, also referred to as the volatile signature, is an informative diagnostic tool for cork quality and sensory qualification. Analytical methods for the study of volatiles rely on gas chromatography with mass spectrometry detection (GC-MS). GC-MS data is combined with sensory evaluation and, in some cases, with olfactometric (GC-O) analysis [9,13,14]. The isolation of the compounds can be performed directly from the cork samples or from the aqueous macerates [10,15,16]. In the latter case, the procedure employed consists in the simulation of the interaction of stoppers with wine under specific maceration conditions and using water or ethanol:water mixtures as matrices [17]. For the preconcentration of volatile molecules, the most commonly used techniques are static headspace (SHS), multiple HS, dynamic HS, headspace sorptive extraction (HSSE), solid phase extraction (SPE) and solid phase microextraction (SPME) [9,10,11,18,19]. The ideal method should have a high capacity and high extraction efficiency, no saturation, moderate selectivity, be easy to automate and not be time-consuming. SPME has been extensively employed in the field of aroma analysis because of its advantages: easy automation, high preconcentration factor, and the possibility of selecting the most appropriate coating for the intended application [20].

In general, the chemical composition of the volatile signature is rather complex. A few volatiles in cork present specific odor descriptors and low odor thresholds. The compound 2,4,6-tricholoranisole (TCA) is undoubtedly the one that has been more extensively studied [18,21], for two main reasons: firstly, it is responsible for the musty, or moldy, aroma, a negative sensory attribute that can be transferred to the wine; and secondly, TCA bears an extremely low sensory threshold, at around 1 ng L^−1^ in water [21]. Other compounds, for example 2,4,6-tribromoanisole, 1-octen-3-one, geosmin, methylisoborneol, 2,3-dimethyl-5-methoxypyrazine and guaiacol, have also been described as contributing to the musty/moldy aroma in corks, but they are rarely found in cork macerates, with the exception of guaiacol [10,22]. Although some of these compounds are endogenous to cork, contamination from external sources has been described, especially in the case of chlorophenols, which were extensively used as pesticides. The transformation of chlorophenols into chloroanisoles as a detoxification mechanism has been demonstrated [23].

The presence of TCA in cork stoppers constitutes one of the most important problems that the cork industry faces, since it causes significant economic losses and can damage the reputation of a cellar. Cork industries are continuously improving their production processes by applying different treatments to cork material to remove odorous compounds, seeking the inertness required in the fabrication of wine closures and, very particularly, the elimination of TCA. Some examples are the ROSA technology developed by the AMORIM cork company, which is based on the use of a distillation process with water steam [24,25], different improved washing procedures, extraction using supercritical carbon dioxide, and the application of different types of radiation or electron beam irradiation [26]. Recio et al. [27] have used hydrogen peroxide as an oxidant catalyzed by molybdate ions in alkaline conditions for the efficient destruction of TCA and pentachloroanisole. Vlachos et al. [28] recommend ozone and/or sterilizing gases, such as steam, via the sequential application of pulsed vacuum-pressure cycles. The most common industrial treatments include temperature and humidity cycles, although the precise characteristics and experimental conditions are not in the public domain. The positive effect of such treatments is a reduction in the TCA levels in cork, which are usually verified at the quality control laboratories of the cork producer. However, other unquantified changes in the cork material may also be expected, such as the modification of the naturally present diversity of microorganisms, changes in the humidity and other physical properties of the material, and the modification of the volatile signature of corks, as determined by sensory analysis.

A systematic strategy for the untargeted investigation of the volatile signature of cork can be applied based on the multivariate analysis of either chromatographic data or data from sensor arrays (an electronic nose) [29]. Boudaoud et al. [30] have studied the volatile signature of cork based on chemometric methods and dynamic headspace (DHS) coupled to mass spectrometry with and without chromatographic separation. Here, we present for the first time the superposing significant interaction rules (SSIR) technique [31] for this purpose. SSIR is a combinatorial procedure which deals with in situ generated rules consisting of the combination of chromatographic variables (pairs of fragment mass and retention time ranges) that probabilistically correlate with a binary classification of the samples. The counting of the variables involved in the most significant rules allows for the identification of relevant fragment masses and times which are presumably responsible for the classification of the samples. From the probabilistic point of view, it is expected that these identified masses are related to the eliminated or newly originated molecules.

The main goal of this study, therefore, is to investigate the possibility of using SSIR together with HS-SPME/GC-MS data for the untargeted analysis of cork volatile signatures, with a special focus on the changes produced when cork samples are submitted to a washing cycle. More specifically, we make an attempt to understand the chemical effect produced by washing cycles on cork barks.

## 2. Materials and Methods 

### 2.1. Reagents and Solutions

The pure standards of compounds listed in Table 1 were all purchased from Sigma. Deuterium-labelled 2,4,6-trichloroanisole (d5-TCA) was used as the internal standard (IS). These compounds were employed to confirm the identification of the peaks observed in the chromatograms.

Individual stock solutions of about 500–3000 mg L^−1^ were prepared by weight in methanol and stored at 4 °C. Mixed working solutions were made by diluting the required volumes of the intermediate solutions (prepared from stock solutions, also in methanol) with ultrapure water. In Table 1, the selected concentrations for the working solutions are shown.

Sodium chloride (99.9%) and HPLC-gradient grade methanol were supplied by Carlo-Erba Reagents. Ultrapure water from a Milli-Q Plus water purification system (Millipore Ibérica) was used.

### 2.2. Headspace Solid-Phase Microextraction Procedure

HS-SPME experiments were manually performed using a 50/30 μm divinylbenzene/Carboxen/polydimethylsiloxane (DVB/CAR/PDMS) fiber from Supelco. Before use, the fiber was conditioned according to the manufacturer’s instructions to remove contaminants and stabilize the solid phase.

Five mL of the sample solution were placed in 15 mL amber glass vials containing 1.2 g of NaCl. Next, 100 µL of the IS solution in methanol (3.2 µgL^−1^) was added. Finally, the vials were closed and introduced to the water bath. The fiber was exposed at 50 °C for 30 min to the headspace above the aqueous solution. Constant stirring was applied during the extraction process. After the completion of the sampling, the fiber was pulled into the needle and the SPME device was removed from the vial and inserted into the injection port of the GC for thermal desorption and analysis.

### 2.3. Equipment and Chromatographic Conditions

Gas chromatographic analyses were performed with a Trace GC 2000 coupled to a PolarisQ ion trap mass spectrometer detector (Thermo Scientific, Waltham, MA, USA). A TG-5SIL MS capillary column (30 m × 0.25 mm i.d.; 0.25 μm film thickness) (Thermo Scientific) was used, and the carrier gas was 99.9990% pure helium (Abelló) at a constant inlet flow rate of 1 mL min^−1^. The split/splitless injection port was operated in splitless mode (maintained for 5 min) at 250 °C.

The oven temperature program started at 40 °C, held for 5 min, increased up to 100 °C at 8 °C/min, increased up to 170 at 5 °C/min, finally increased up to 270 at 15 °C/min and held for 2 min; the total run time was 35.0 min. The transfer line temperature was set at 270 °C, and the ion source temperature was set at 225 °C. MS analyses were conducted in scan mode with two m/z intervals: from 40 to 250 amu (from min 3 to 19 min) and from 100 to 350 amu (from 19 min to the end of the chromatographic run). Ionization was performed in the electron impact mode at 70 eV. The acquisition of chromatographic data was performed using Xcalibur 1.4 software (Thermo Scientific). Table 1 shows the list of the target compounds, their retention times and their qualifier ions.

### 2.4. Cork Samples and Preparation of Cork Macerates

The 56 samples consisted of cork discs obtained before (28 samples) and after (28 samples) the temperature and humidity cycles addressed to eliminate TCA (see supplementary Table S1). According to the information provided by the cork stopper producer, the TCA level was reduced to <0.5 ng L^−1^ after the treatment. The sensory evaluation performed by a panel in the company concluded that an increase in vanilla, biscuit and sweet notes was detected after the washing cycle.

The cork macerates were obtained by contacting 6 discs (about 6 g of cork) with 100 mL of ultrapure water at 50 °C for 48 h. The solid parts were then separated and the aqueous cork macerate was freeze-dried until the analysis was performed. 

### 2.5. Dataset and Data Processing Algorithms

Firstly, targeted analysis was conducted by exploring the chromatographic results, and peak areas were recorded using the ions in bold in Table 1. Relative peak areas (the area of the compound divided by the area of the IS) were calculated. Principal component analysis (PCA) was performed using Minitab [32] and in-house software.

Secondly, an untargeted approach was implemented. The signal (total ion current) was recorded and discrete values were obtained in the form of a data set where the contributions of all m/z fragments at each time were obtained. This information was easily obtained using Xcalibur software. After a preliminary exploration, the time range considered was from 6 to 17 min, and only ions with amu 49–170.5 were considered.

The procedure considered to extract the relevant descriptors capable of classifying the samples was the superposing significant interaction rules (SSIR) method. This procedure was originally designed as a variable selector and applied in the field of structure–activity relationships. The algorithm allowed the modelling of binding affinities [31], and the classification of former inhibitors of the HIV-1 reverse transcriptase [33] and a family of substituted peptides [34]. The procedure is related to the design of experiments field, and has been applied to obtain fast qualitative preliminary results before the development of a full design [35]. This method is presented here for a chromatographic application for the first time.

## 3. Results and Discussion

### 3.1. Targeted Analysis and First Attempt to Evaluate the Impact of Cork Treatment Using Principal Component Analysis

As already mentioned in Section 2.4, the aqueous cork macerates were analyzed by a sensory panel, and the main conclusion was an increase in biscuit and sweet aromatic notes in the treated samples.

A list of compounds contributing to the volatile signature of cork was established from a survey of data from the literature (Table 1), and a solution containing all the compounds and the IS was extracted and analyzed by the proposed method (Section 2.2). Retention times were collected and used for the identification of the compounds in the samples. 

Some of the target compounds were identified in the set of studied samples both before and after the washing cycle. It was expected that the washing cycle would contribute to the elimination not only of TCA but also of other volatiles present in the cork disc. An inspection of the area ratios (see supplementary Table S1) has shown that the maximum signal was reduced for some of the volatiles, including TCA, but no clear trend in the decrease in the values for washed cork samples was observed. 

**Figure 1:**
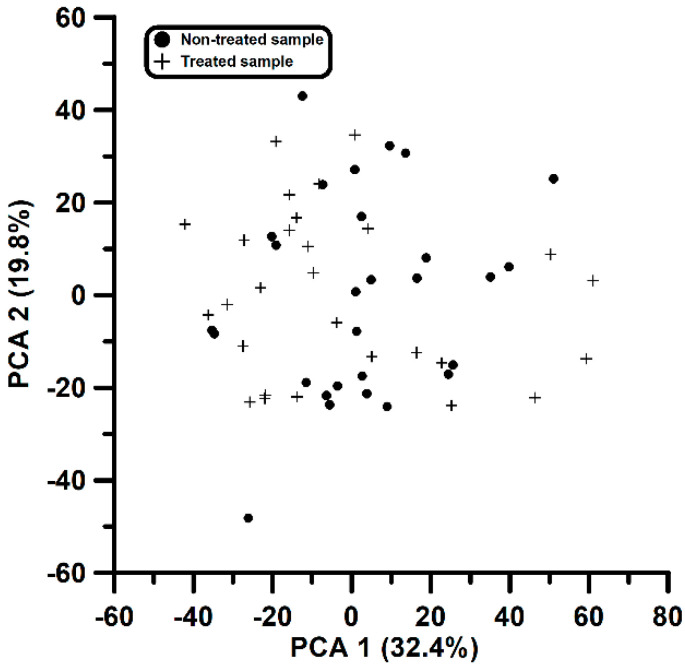
Typical results obtained during the first study by means of PCA. No clear separation of samples (non-treated 0 and treated 1) was obtained.

Further analysis was performed using PCA, giving a plot as shown in Figure 1. No useful information could be extracted. For instance, in Figure 1 the first two components only collect 32.4% and 19.8% of the total variance, respectively. The first principal component shows maximum squared loadings (ranging from 0.16 up to 0.29) for four compounds (eucalyptol, fenchol, camphor and αterpineol), whereas the second component is mainly defined by guaiacol and borneol (squared loadings of 0.44 and 0.37, respectively). The contribution of TCA in both components is almost zero. Similar results to those shown in Figure 1 did not help to identify a compound or set of compounds that were responsible for the differences found in the olfactory evaluation between treated and non-treated samples.

It was not possible to evaluate the influence of the treatment and to explain the differences observed in the sensory analysis from this first attempt. The need is therefore seen for a different, objective approach to the evaluation the effect of the treatment and for the identification of a set of compounds that can explain the olfactory characteristics described by the panel.

### 3.2. Data Processing Results Obtained with SSIR

**Figure 2:**
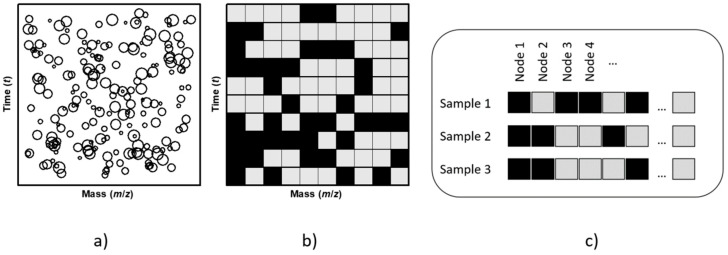
Schematic representation for a sample of the variables and levels grid definition needed to apply the superposing significant interaction rules (SSIR) method: (**a**) a bubble graph representation of the original peak signals; (**b**) after gridding, each node is ultimately assigned to a binary label: low level (grey) or high level (black); (**c**) representation of the discretized grid variables and the respective low and high level series for three samples. Each sample bears its own distribution of labels (codification) along the grid. See text for more details.

The set of 56 samples was dichotomized into two classes: treated and non-treated. The bulk chromatographic raw data file was obtained using the file converter tool of the Xcalibur software. For each sample, all the time and mass registers were reorganized in a rectangular grid of time and mass combination (see the intensities represented in the schematic bubble chart of Figure 2a). A first calculation using the whole data in the time domain between 3 and 19 min and mass fragments 40–350 was used to confirm that no variable was selected before 6 min, and that the ions of mass 210 and 212 attached to TCA (see Table 1) did not appear at a time greater than 18 min.

Hence, in order to reduce the search space and exclude d5-TCA, which was used as an internal standard, the time range was set from 6 to 17 min. The corresponding time partitions were 20 s (1/3 min) wide, resulting in 33 intervals. In turn, the range for the permitted m/z variable values was set from 49 to 170.5 amu. In the case of the mass interval, this was partitioned into 243 intervals (i.e., in steps of 0.5 amu), centered at half integer and integer mass values. Therefore, the original rectangular grid was made up of 33 × 243 = 8019 nodes for each sample, with each node being the combination of two intervals (time and ion mass). A symbolic representation (in a simple schematic 10 × 10 grid) of the time and mass variable combinations is shown in Figure 2b.

All the intensity registers were normalized by re-scaling and setting the mean of intensities to an arbitrary value of 1000 units per sample. All these intensity values were then added into the corresponding nodes of Figure 2b, according to the defined time and mass intervals. Therefore, the available data descriptors per sample are a two-dimensional grid collecting a sum of intensities in each node.

The SSIR method deals only with categorical variables. For the sake of simplicity, only binary variables have been considered here, as it was decided to dichotomize the values of the collected intensities in each node. The top 1% percentile values constituted the high level (black squares in Figure 2b,c), and the remaining values constituted the low level (grey squares). In effect, each sample has been codified by a string of levels (Figure 2c). The goal is to select those grid nodes (i.e., combinations of time and mass intervals) that correlate with the previously defined dichotomization label of the samples.

Figure 2c shows a simplified representation of the discretized grid variables and the codification series of low and high binary levels for some samples (horizontal readings). Particular possibilities were considered. Firstly, some nodes can exhibit a constant state over all the samples, i.e., they are always present the same level feature (see, for instance, the vertical readings along the first and last drawn nodes in Figure 2c). These types of nodes are not useful at all and were discarded. Of the 8019 dichotomized nodes, only 299 change their low or high condition over the samples. Secondly, some of the remaining nodes could present a degeneration, i.e., the same pattern variation along the samples, as is shown schematically for nodes 3 and 4 in Figure 2c. These degenerated nodes are equivalent and redundant. In these cases, only a single node is passed to the SSIR procedure, together with the individual nodes which are not degenerated (all these nodes are called representative nodes or, simply, representatives). Of the previously mentioned 299 non-constant nodes, 237 were selected as representatives. The SSIR procedure detects which representative binary nodes are relevant to classify the samples. At the end of the calculation, the final reported variables are those attached to all the selected nodes and also the represented ones, if any degeneration has occurred.

The SSIR algorithm generates rules. A rule is the combination of several levels of distinct nodes. Normally, and for the sake of simplicity, only order 2 and 3 rules are considered when applying SSIR. This is the same as occurs within the design of experiments theory: usually contributions and interactions of two or, at most, three factors are able to explain the relevant variables governing the experiment. Despite finding that order 2 rules were able to detect the relevant variables in this chromatographic problem, surprisingly, the simplest rules of order one performed equally well. This implies that all of the 237 discretized variables were inspected alone, one at a time. A total of 11 representative nodes exhibited a *p*-value less than or equal to the threshold value of 0.0005 (see below). As was expected, all intervals centered on half integer values were automatically discarded by the procedure. The total number of represented variables is 13. Table 2 lists the corresponding associated intervals of the selected variables (see supplementary material Algorithm S2 for details of the ranking procedure).

For each variable, a reckoning was performed to look for the number of high and low levels found along the two classes of samples. Table 3 presents the relevant data. The values for high and low levels of the same kind of sample (either treated or non-treated) are complementary, with the total sum being the constant of 28 samples. Visually, the asymmetric distribution of values along the columns of high and low variable levels shows classification tendencies. For instance, variables 2 and 7 are found at the high level in all the treated samples, but also in many non-treated ones (5 and 15, respectively). Remarkably, variables 3 and 4 are found at the high level in 23 of the treated samples and in none of the non-treated ones. On the other hand, variables 3, 4, 6, 9, 10 and 11 are present at a low level in all the non-treated samples, and the first two of them are present at a low level in only five treated samples. Variable 12 is present in its low level in all the treated samples.

Similarly, variable 13 is present at a low level in all of the treated samples but one. Variables 12 and 13 can be associated with the disappearance of a certain compound or compounds. This is also concurrent with the fact that the mean peak areas of these variables among treated samples is less than that of the non-treated ones. These numerical distributions allow the significance attached to each selected variable to be evaluated (*p*-value). The probabilistic calculation of the *p*-value was performed using equations 6 and 7 of reference [31].

The key idea of the SSIR method is that each variable acts as an a priori random extractor and classifier of samples. The *p*-value is then used to determine how difficult it is to find a certain number of treated samples within the set selected by the variable. In this study, each time the *p*-value is equal to or less than the predefined threshold, the variable was selected for classification purposes, as it is expected to correlate with the dichotomic classification of the samples.

It has been found by inspection that, for all but the last two variables, there is a correlation with the increase in the chromatographic mean intensities of the treated samples with respect to the non-treated ones. Hence, the related ions are to be attached to compounds that were generated after sample treatment. This was surprising, as the treatment (temperature and washing cycles) was conducted with the aim of removing compounds. With regards to the last two variables in Table 2, the mean cumulative areas among treated samples is about half the areas found among the non-treated ones. These ions can be attributed to the process of compound removal.

**Figure 3:**
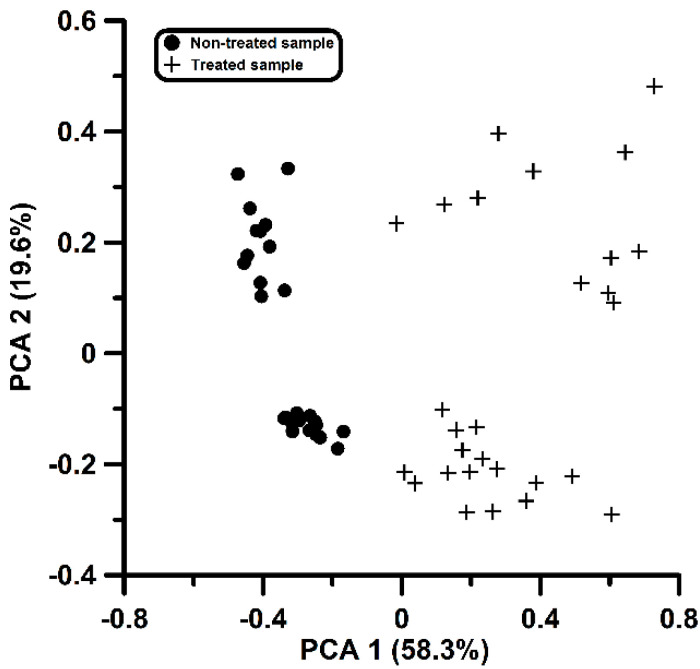
PCA representation of the treated and non-treated samples from the selected variables by SSIR. See text for more details.

The selected variables in Table 3 were considered for modelling purposes. The variables were entered as descriptors in a PCA calculation using the original sum of collected peak intensities found before the dichotomization as the descriptor value (see supplementary Table S3). The scores map generated is depicted in Figure 3. The first principal component retains 58.3% of the variance, and the second, 19.6%. This constitutes an important increase with respect to the values found in Figure 1. Furthermore, it is clearly seen that the first component alone acts as a discriminant function for the classification of both types of samples. The loadings of the two first components are given in the last column in Table 2. The loadings along the first principal component are similar, and almost all the variables contribute equally. The last two variables are associated with a negative contribution. This is not surprising because these variables are associated with a possible disappearance of a compound, as has been mentioned above. The behavior of variables 11 and 12 is worth noting: both are attached to the same mass of 51 units, but the first appears in treated samples while the last one seems to disappear after the washing treatment. Despite the fragment mass being the same, the origin must be attributed to distinct compounds, given that they correspond to different time intervals (i.e., retention times).

### 3.3. Discrimination and Prediction

As stated above, the first principal component described above constitutes a valid variable to discriminate samples. The discriminant model correctly classified all but one of the samples. Thus, the sensitivity of the model was about 96% and the specificity was 100%. In order to test the robustness of such a model, a leave-one-out cross-validation calculation was conducted. The procedure consisted of the elimination of one sample at a time and then redoing all of the calculations with the remaining 55 (i.e., reading all the bulk chromatographic files from scratch, defining the grid and dichotomizing it, constructing the SSIR model, performing the PCA calculation and deriving the linear discriminant function). The discriminant function was then applied to the removed sample in order to predict whether it was treated or non-treated. All of the 56 samples passed their own individual cross-validation tests, and for all but two of them the prediction was correct (96% performance). Additionally, discriminant functions involving 2 to 5 of the first principal components were also considered in all the leave-one-out procedures. When the model involved the two first principal components, only one classification error was found. The models involving 3 to 5 principal components correctly classified all of the samples.

### 3.4. Identification of the Compounds Responsible for Group Separation

**Figure 4:**
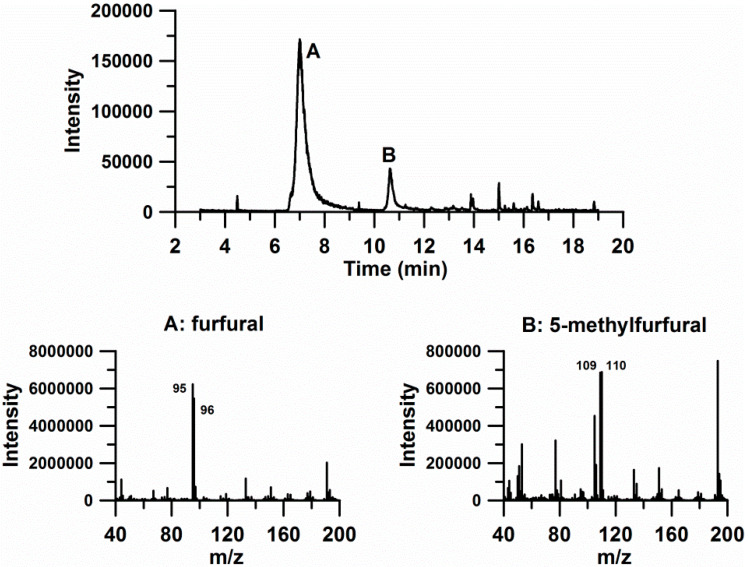
XIC of sample ‘mostra46R-1xCD’. The ions selected were 51+67+78+95+96+97+109+110. The mass spectra of the two main peaks, with identifications according to the NIST library, are shown below.

The acquired chromatograms were inspected, taking into consideration the information obtained from the SIRR procedure and Table 2. From the table, three specific time intervals can be distinguished, corresponding to different compounds eluting out of the chromatographic column: from 6.7 to 7.7 min (with ions 51, 67, 95, 96 and 97), from 10.3 to 11.0 min (with ions 109 and 110) and from 8.7 to 9.3 min (with ions 51 and 78). The three intervals correspond to variables 5, 7, 8 and 11 (first interval), variables 3, 4, 9 and 10 (second interval), and variables 12 and 13 (third interval), as shown in Table 2. An inspection of the NIST library allows a tentative identification of the compounds for the two first time intervals, namely furfural and 5-methylfurfural (see Figure 4). The identity of the compounds was verified by injecting pure standards. Variables of the third interval could not be identified despite them possibly playing a relevant role in the treatment; these are the only variables associated to compounds that presumably disappear or are removed due to the washing cycles. Taking into account the results shown in Figure 3, we can conclude that the three compounds are responsible for the separation of the 56 cork samples into the treated and non-treated groups, which is achieved by the untargeted analysis of chromatographic data without the possibly subjective contribution of human perception. 

The presence of various furan derivatives in cork samples has already been described [11,36,37,38]. The authors related the presence of various furan derivatives to carbohydrate degradation (Maillard-type and acid-catalyzed sugar degradation). This suggests that the thermal treatment applied to corks intended to reduce the TCA content may have the parallel effect of favoring an increase in the concentration of furans. In fact, furfural and 5-methylfurfural were found to be present in wines aged in barrels. Furfural compounds are formed by thermolysis of the cellulose and hemicelluloses, together with Maillard reactions that take place during cooperage [39]. Moreover, furfural and 5-methylfurfural are described as having an almond-type aroma, which agrees with the sweet notes identified in treated cork by the sensory panel.

Boudaoud et al. [30] demonstrate a DHS-MS method in conjunction with multivariate analysis as a promising and simple solution to determine the origin of cork. In the present study, the HS-SPME/GC-MS-SSIR method has shown itself to be a useful tool not only in distinguishing between treated and non-treated cork samples but also in identifying the volatile compounds that are responsible for the sensory changes produced as a result of the treatments. The industrial relevance of these findings is evident.

## 4. Conclusions

Given the challenging problem of sample classification for which traditional numerical treatment of chromatographic data was inconclusive, it has been demonstrated that the application of the SSIR procedure leads to the identification of a set of 13 relevant variables. These variables allowed for a PCA and DA classification with good figures of merit. The inspection in detail of these variables in the original chromatographs leads to the conclusion that three compounds are responsible for the separation of the 56 cork samples into the treated and non-treated groups presenting different sensory descriptors. Additionally, despite the treatment of samples being conducted in order to remove certain compounds, thanks to the proposed methodology, evidence has been found that, on the contrary, some new compounds, i.e., furfural and 5-methylfurfural, were also possibly generated by the treatment itself.

## Figures and Tables

**Table 1:** Compounds used for target analysis of cork volatiles: retention time, m/z fragments used for the identification and concentration of the compounds in the working solution prepared to confirm the identification.

Compound	Retention Time (min)	m/z ^1^	Concentration (µg L^−1^)
1,8-cineol (eucalyptol)	12.33	**81,**108**,**139**,**154	3.95
MDMP ^2^	12.66	**137**	0.46
IPMP ^2^	13.55	**137**	0.38
Guaiacol	13.51	**81,109,124**	19.58
(±)-linalool	13.88	**71,93,**121	3.33
(+)-fenchol	14.33	**81**	5.26
1,2-dimethoxy benzene (veratrol)	14.84	**95,123,138**	15.62
Camphor	15.00	**95**	3.66
Sec-IBMP ^2^	15.42	**124,138**	0.68
(-)-borneol	15.6	**95**	4.25
IBMP ^2^	15.65	**124**	21.12
Menthol	15.71	**95**	3.82
Methylisoborneol	16.02	**95,108**	0.57
α-terpineol	16.13	**93,121,**136	5.54
Benzothiazole	16.86	**108,135**	21.89
TCA	19.30	**210,212**	0.01
d5-TCA	19.22	**215,217**	0.06
Geosmin	21.50	**112,**125	0.6

^1^ m/z in bold are used for quantification. ^2^ MDMP: 2,3-dimethyl 5-methoxy-pyrazine; IPMP: isopropyl methoxy pyrazine; IBMP: isobutyl methoxy pyrazine.

**Table 2:** Selected variables by SSIR after the binary codification of the signals.

Variable #	Time Interval (min)	m/z Interval (amu) ^1^	1st PCA Loading in Figure 3	2nd PCA Loading in Figure 3
1	[7.3, 7.7)	95	0.335	−0.106
2	[6.7, 7.0)	95	0.225	0.286
3	[10.3, 10.7)	110	0.341	0.194
4	[10.3, 10.7)	109	0.332	0.221
5	[7.0, 7.3)	97	0.315	−0.058
6	[7.0, 7.3)	67	0.294	0.166
7	[6.7, 7.0)	96	0.249	0.198
8	[7.3, 7.7)	96	0.299	−0.243
9	[10.7, 11.0)	109	0.285	−0.217
10	[10.7, 11.0)	110	0.272	−0.286
11	[7.0, 7.3)	51	0.308	0.237
12	[8.7, 9.0)	51	−0.106	0.497
13	[9.0, 9.3)	78	−0.111	0.509

^1^ The interval center is indicated. The interval has a radius of 0.25 amu.

**Table 3:** Reckoning of high or low levels for dichotomic selected variables of the treated and non-treated samples.

Variable #	High Level		Low Level		*p*-Value ^1^
	Non-Treated Samples	Treated Samples	Non-Treated Samples	Treated Samples	
1	2	27	26	1	1.4·10^−12^
2	5	28	23	0	3.1·10^−11^
3	0	23	28	5	3.1·10^−11^
4	0	23	28	5	3.1·10^−11^
5	4	25	24	3	9.3·10^−9^
6	0	16	28	12	7.3·10^−7^
7	15	28	13	0	2.0·10^−5^
8	14	27	14	1	7.1·10^−5^
9	0	11	28	17	1.4·10^−4^
10	0	11	28	17	1.4·10^−4^
11	0	10	28	18	3.7·10^−4^
12	10	0	18	28	3.7·10^−4^
13	12	1	16	27	4.7·10^−4^

^1^ According to the formulation given in the text. The total number of samples is 56 of which 28 are treated and 28 are non-treated.

## Supplementary Materials

The following are available online at https://www.mdpi.com/2218-273X/10/6/896/s1. Table S1: List of samples and the condition of being treated (1) or non-treated (0). Area ratios for each target compound used in the first manual inspection and in the first PCA analysis, conducting to Figure 1; Algorithm S2: Description of the SSIR algorithm adapted to dichotomic variables selection; Table S3: List of samples and the condition of being treated (1) or non-treated (2). Peak intensities collected for each sample at the 13 variables (Table 2) selected by SSIR procedure. The PCA of this matrix conducted to Figure 3.
