# Supplementary material for: Investigation of Volatiles in Cork Samples Using Chromatographic Data and the Superposing Significant Interaction Rules (SSIR) Chemometric Tool
Source: Biomolecules. 2020 Jun 11;10(6):896. doi: 10.3390/biom10060896 (PMC7355702; doi:10.3390/biom10060896)
Supplement: Supplementary file 1 [file biomolecules-10-00896-s001.zip › biomolecules-788457-supplementary new/SSIR-Extension_S2.pdf]

Investigation of volatiles in cork samples using chromatographic data and the superposing significant interaction rules (SSIR) chemometric tool

Emili Besalú, Chantal Prat and Enriqueta Anticó

Appendix S2: Superposing Significant Interaction Rules (SSIR) algorithm

This appendix briefly describes the foundation of SSIR method when employed as a variable selector. The procedure was originally designed into the field of combinatorial chemistry and QSAR but here the text has been adapted for the treatment of categorized variables that describe a set of available samples. In general, each variable can be partitioned into an arbitrary number of levels but in this work each variable was dichotomized and presents only two states: high (H) or low (L). In present application, the goal of SSIR is to find combinations of variable levels that correlate with a particular binary property of the samples: being a treated cork sample or a non-treated one. In the cited references of SSIR (see main text), the reader can find more details about the method nuances.

Descriptor variables

Consider a set of samples described by *n* categorized variables, each one presenting two possible levels (H or L). Each sample is being represented by a particular *n*-tuple that collects the particular levels that acquires each variable. For instance, if each sample is represented by 4 variables, its description can be the *HLLH* string (level *H* for first descriptor, level *L* for the second, and so on) or the *LLHL* one. In this work, and after data filtering, each sample has been described by 237 non-redundant representative dichotomized variables (see main text).

Numerical procedure

SSIR method consists into loop over combinations of *k*=1, 2, 3, ... variables, and for each combination loop over all the possible variations of levels. Each sequence of *k* level variables conforms a *rule* of order *k*. It is said that each rule *condenses* the particular subset of samples that conform with the selected variable levels (see the example below). Across the subset of condensed samples, some of them will be treated ones and some will not. SSIR procedure evaluates the rules (as explained below, comparing the proportion of treated/non-treated with the total proportion found across all the available samples) and selects some of them in order to correlate some level variables with the samples property.

Each rule is evaluated from a probabilistic point of view, according to the number of condensed samples which are of interest. Due to the fact that the sample classification property is always binary, the elements labeled as being “of interest” can be either the treated ones or the non-treated ones. The evaluation is made by calculating an attached *p*-value per rule. The rules exhibiting a low *p*-value are said to be significant and the involved

variable levels receive cumulative votes. It is expected that the higher the number of votes a variable level collects, the higher is the correlation of this level with the subset of interest. For the particular application of this work, and taking into account that the variables are dichotomic, it is enough to keep the reckoning of the variables (not the levels) that entered in significant rules.

Probabilistic significance attached to a variable

Let us suppose that we have to classify a set of *a* samples, and *b* of them are labeled as being of interest (either treated ones or non-treated ones in the main text). Then, if a particular rule condenses *c* of those samples and *d* of them are also of interest, one can ask for the conditional probability of this event:

$$P\left(d,c\,|\,b,a\right)=\frac{\binom{b}{d}\binom{a-b}{c-d}}{\binom{a}{c}} \quad \text{with } d\le c\le a \text{ and } d\le b\le a.$$

This probability is equivalent to the one concerning the random selection (pick-up) of *c* samples and, a posteriori, performing the reckoning of how many are of interest. Hence, a rule can be understood as a random selector.

The *p*-value attached to the rule is obtained from the cumulated probabilities that the rule condenses *d* or more (*d*+) structures of interest. In other words, given that the set of *a* samples presents *b* of interest, then the conditional probability of finding *d* or more of interest when randomly selecting *c* is:

$$p\left(d+,c\,|\,b,a\right)=p\left(d:min\left(b,c\right),c\,|\,b,a\right)=\sum_{i=d}^{min(b,c)}P\left(i,c\,|\,b,a\right)=1-\sum_{i=max(0,c+b-a)}^{d-1}P\left(i,c\,|\,b,a\right).$$

Rules generation

SSIR algorithm computes the respective *p*-value for all the possible rules (of pre-selected orders) definable in the samples set. In general, if the samples are described by *n* variables, there is a total of $C(n,k)=\binom{n}{k}$ combinations of groups of *k* variables. Each combination generates a fixed number of rules of order *k*. If each variable presents 2 levels, each combination of order *k* generates 2^k rules. It is common to explore only low rule orders, as it is justified in the literature.

From the algorithmic point of view, the systematic generation of rules is performed nesting two combinatorial entities (see below the SSIR basic algorithm). The first one generates combinations among *k* variables in order to set up the rule order, and the second one generates the permutations with repetition among the levels of the previously selected variables. In the literature it is described how a third combinatorial entity can be also

considered in order to define the so-called level negations, but this is not treated here because this entity does not apply for dichotomized variables.

Algorithm

The following algorithm constitutes the basic implementation of SSIR employed to rank a series of dichotomic variables. The procedure generates the rules, evaluates the corresponding *p*-values, selects the variables involved in rules presenting a *p*-value below the threshold defined per each order and, finally, sorts the variables according to the number of times the variable was employed in significant rules. It is assumed that the first ranked variables are more suitable to classify the samples (for instance, using PCA, Discriminant analysis or other linear or non-linear techniques).

Algorithm: Basic SSIR procedure for variable sorting (dichotomic variables).

1. Samples data information:
    1.1. Set the number of samples: *a*.
    1.2. Partition the sample set into two subsets: the ones of interest and the ones of non-interest. Count the total number of samples of interest: *b*.
    1.3. Set the number of dichotomic variables describing each sample: *n*.
    1.4. Set variable counters to zero: $V(v)=0$ for v=1,n.

2. Set the range of rule orders to be explored: $[k_i , k_f]$ where $1 \leq k_i \leq k_f \leq n$.

3. Set the threshold *p*-value per rule order: $p(k)$, $k = k_i , ... , k_f$.

4. Generate rules and explore the probabilistically relevant ones:
    Loop for $k = k_i , k_f$. Loop over rule orders.
        Loop: For each rule order generate the $C(n,k)$ combinations of variable selections.
            Loop: Generate the 2^k variations among the levels of the selected variables.
                Each particular variation among combined variables defines a rule: *r*.
                For each rule *r*:
                    Count how many samples are condensed by the rule: *c*.
                    Count how many of these samples are of interest: *d*.
                    If $p(d+,c|b,a) \leq p(k)$ then the rule is a significant one and then
                        For v=1,k: Loop over the involved variables in rule *r*
                            $V(M_{v,r})=V(M_{v,r})+1$: Increment the counter of variable $M_{v,r}$ (*v*-th variable in rule *r*).
                        End Loop
                    end if
            End Loop of variations.
        End Loop of combinations.
    End Loop over rule orders.

5. The original set of variables is ranked. Sort variables according to counters: $V(1) \geq V(2) \geq ... \geq V(n)$.

Alternatively, if necessary, along the algorithm instead of reckoning variables present in significant rules, the $V(v)$ counters, it is also possible to cumulate the votes along the levels of these variables: instead use $L(V,v)$ counters. In our work we were interested in detect relevant variables but the algorithm can be used to go a step beyond and detect the relevant level(s) of each variable. This is particularly useful if some of the variables present more than two levels.

A simple example

A very simple artificial example follows in order to show how SSIR algorithm operates. Let us suppose we have a set of a=100 samples and that b=3 of them are samples classified as being of interest. The samples are described by n=4 binary variables. Each descriptor exhibits the value of H or L. The following table collects all the data:

Sample #	Is of interest	Variable 1	Variable 2	Variable 3	Variable 4
1	Yes	H	L	H	L
2	Yes	H	H	L	H
3	Yes	H	H	H	L
4	No	H	L	L	L
5	No	H	H	L	H
6 – 97	No	L	L	L	L
98	No	L	L	L	H
99	No	L	H	H	L
100	No	H	L	L	H

For sake of simplicity, the samples 6–97 are of non-interest and all the respective descriptors exhibit the level L at each variable.

If rules of order k=2 are being considered, these rules arise from the $C(n,k)=C(4,2)=6$ possible combinations of variables. One of these combinations involves variables 1 and 2. As each variable exhibits two levels, there are a total of $2^k=4$ possible rules obtained with the combination of variables 1 and 2. The rules arising from this particular combination of variables and levels are {1:L,2:L}, {1:L,2:H}, {1:H,2:L}, and {1:H,2:H}. That is: variable 1 at level L and variable 2 at level L; variable 1 at level L and variable 2 at level H; and so on. Take for instance the last rule {1:H,2:H}. The number of samples that are condensed by this rule is c=3 (i.e., only samples # 2, 3, and 5 fulfill the conditions of the rule). Among this subset, the number of condensed samples that are of interest is d=2 (samples # 2 and 3). This event is attached to a p-value of

$$p(2+,3|3,100) = P(2,3|3,100) + P(3,3|3,100) \approx 0.001800 + 0.000006 \approx 0.001806.$$

If it is considered that this p-value is small enough, the rule becomes significant. In this case the involved variables (variables # 1 and 2 in this example) will increment their counters in one unit. As said, it is also possible to increment the counters attached to the levels present in the rule.

After all the possible rules are generated (considering all the combinations and all the variations) and the eventual counters incremented, the variables (or levels) collecting the greatest number of votes are to be ranked first.
