# Supplementary material for: Investigation of Volatiles in Cork Samples Using Chromatographic Data and the Superposing Significant Interaction Rules (SSIR) Chemometric Tool
Source: Biomolecules. 2020 Jun 11;10(6):896. doi: 10.3390/biom10060896 (PMC7355702; doi:10.3390/biom10060896)
Supplement: Supplementary file 1 [file biomolecules-10-00896-s001.zip › biomolecules-788457-supplementary new/Table_S1.pdf]

Table S1. List of samples and the condition of being treated (1) or non-treated (0).
Area ratios for each target compound used in the first manual inspection and in the first PCA analysis, conducting to Figure 1.

Treated?	Sample #	Sample Name	eucalyptol	guaiacol	fenchol	veratrole	camphor	borneol	alfa-terpineo	enzothiazol	TCA
**0**	**1**	**mostra1**	0.57588	0.11150	0.05935	0.23544	1.35635	0.10809	0.95009	0.21555	0.09660
**0**	**2**	**mostra2**	0.24821	0.10870	0.06367	0.15707	1.91469	0.24704	1.22721	0.21812	0.08790
**0**	**3**	**mostra3**	0.50209	0.06357	0.06154	0.17601	1.51853	0.13519	1.06950	0.21055	0.09679
**0**	**4**	**mostra4**	0.86369	0.09892	0.08091	0.26488	2.04501	0.23370	1.85446	0.27210	0.10871
**0**	**5**	**mostra5**	0.76147	0.13796	0.05898	0.09026	1.76323	0.13631	1.10205	11.15134	0.10056
**0**	**6**	**mostra6**	0.91331	0.12535	0.09906	0.16019	2.07963	0.26898	1.41920	12.50908	0.10920
**0**	**7**	**mostra7**	0.59877	0.19397	0.06745	0.16641	1.58457	0.14623	1.28770	5.35578	0.11421
**0**	**8**	**mostra8**	0.43221	0.42509	0.11593	0.39311	2.46742	0.40745	2.51005	0.41660	0.14408
**0**	**9**	**mostra9abans**	0.31215	0.12447	0.07670	0.20843	0.94566	0.07031	0.55576	0.63442	0.14436
**0**	**10**	**mostra10abans**	0.56243	0.08113	0.13819	0.28210	1.43779	0.10135	0.86921	0.97600	0.14482
**0**	**11**	**mostra11abans**	0.39603	0.21715	0.09873	0.17232	0.94467	0.09559	0.58335	4.70218	0.13217
**0**	**12**	**mostra12abans**	0.68349	0.25974	0.09487	0.23746	1.02172	0.07345	0.65759	1.19706	0.11940
**0**	**31**	**mostra121NRNOCD**	0.08362	1.08711	0.09075	0.10769	1.10691	0.47239	0.76960	0.74948	0.06030
**0**	**32**	**mostra121RNOCD**	0.06733	2.26392	0.07678	0.14824	1.13118	0.43788	0.80188	1.19039	0.11706
**0**	**34**	**mostra129NRNOCD**	0.20555	1.07848	0.03392	0.20015	1.24166	0.30579	0.58796	0.65504	0.18826
**0**	**35**	**mostra90NRNOCD**	0.34783	1.67887	0.06514	0.23312	1.98627	0.47018	1.41024	1.16501	0.05047
**0**	**36**	**mostra90RNOCD**	0.41868	1.90816	0.06384	0.50324	1.60150	0.34441	1.13314	0.64640	11.87900
**0**	**37**	**mostra99NRNOCD**	0.55040	0.87796	0.06731	0.33313	1.95998	0.24586	0.57779	0.66149	0.10125
**0**	**38**	**mostra99RNOCD**	0.38102	1.58348	0.09852	0.29480	1.92001	0.48913	1.08807	0.84560	0.38197
**0**	**40**	**mostra46R-NOCD**	0.52547	0.94330	0.14100	0.20100	2.05378	0.59884	3.15605	0.89019	0.09257
**0**	**42**	**mostraCln1NR-NOCD**	0.13340	0.44504	0.03366	0.28538	0.69702	0.14615	0.14107	3.09035	0.06629
**0**	**44**	**mostraCln2NR-NOCD**	0.08002	0.12306	0.03909	0.21980	0.60219	0.21474	0.10873	1.66371	0.09430
**0**	**46**	**mostrad28NR-NOCD**	0.36740	0.53151	0.08475	0.11385	2.05668	0.34031	0.63604	5.64317	0.07780
**0**	**48**	**mostraHDn1HDNR-NOCD**	1.30102	1.18168	0.06280	3.33232	2.14951	0.30330	0.73204	0.60421	0.08545
**0**	**50**	**mostraHDn1TZNR-NOCD**	0.34720	1.30464	0.05000	0.14357	1.06444	0.25147	0.53532	0.90635	0.04272
**0**	**51**	**mostraHDn2HDNR-NOCD**	0.19442	0.87101	0.10100	0.23766	1.55930	0.43269	0.77219	0.59219	0.04797
**0**	**52**	**mostraHDn2TZNR-NOCD**	0.67314	0.81134	0.08069	0.43219	2.11772	0.32425	1.14002	3.62931	0.05250
**0**	**54**	**mostraCCM-NR-NOCD**	0.71754	0.90145	0.08735	0.19615	2.28837	0.39323	0.93660	1.07450	0.06884
**1**	**13**	**mostra1desp**	0.85777	0.30576	0.05780	0.22513	1.61801	0.24226	2.04841	3.96695	0.09894
**1**	**14**	**mostra2desp**	1.25816	0.34826	0.07693	0.53362	2.91567	0.54455	2.83415	0.99716	0.11143
**1**	**15**	**mostra3desp**	0.62074	0.15314	0.05095	0.30176	1.51864	0.21940	1.61639	5.47738	0.32688
**1**	**16**	**mostra4desp**	1.20746	0.23683	0.09460	0.39980	3.03318	0.30107	2.95377	1.80377	0.14101
**1**	**17**	**mostra5desp**	1.36801	0.19311	0.10043	0.20050	2.40644	0.23974	2.28521	0.98585	0.11019
**1**	**18**	**mostra6desp**	0.41738	0.17004	0.06689	0.16263	0.74344	0.06283	0.47235	0.23155	0.14199
**1**	**19**	**mostra7desp**	1.22441	0.34362	0.13086	0.17547	1.81033	0.17339	0.84610	0.29550	0.11445
**1**	**20**	**mostra8desp**	0.98172	0.48481	0.13821	0.24126	1.60709	0.22139	1.00719	0.41959	0.14816
**1**	**21**	**mostra9desp**	0.41734	0.10418	0.05743	0.12328	0.66093	0.06854	0.37368	0.34424	0.10642
**1**	**22**	**mostra10desp**	0.48000	0.09134	0.06248	0.15324	0.70506	0.10968	0.36705	0.34518	0.10036
**1**	**23**	**mostra11desp**	0.50128	0.12628	0.07370	0.19460	0.90287	0.09397	0.67889	0.39946	0.10598
**1**	**24**	**mostra12desp**	0.49560	0.08791	0.09396	0.15170	0.89198	0.36283	0.62304	0.24779	0.36573
**1**	**25**	**mostra121NR**	0.04865	1.13097	0.08921	0.09338	0.96505	0.44267	1.01606	0.47132	0.05260
**1**	**26**	**mostra121R**	0.04501	0.31327	0.04338	0.08101	0.42528	0.25451	0.61807	0.34795	0.09980
**1**	**27**	**mostra90NR**	0.00000	1.34508	0.00000	0.28213	0.48313	0.22178	0.57982	0.73604	0.05693
**1**	**28**	**mostra90R**	0.10666	1.25527	0.07831	0.29605	1.03837	0.57395	1.48560	1.10322	0.05069
**1**	**29**	**mostra99R**	0.09959	1.29892	0.04052	0.39940	0.83175	0.20687	0.63870	0.51499	0.04975
**1**	**30**	**mostra99NR**	0.13034	1.41549	0.05281	0.37080	1.21692	0.33565	0.71233	0.67373	0.05823
**1**	**33**	**mostra129NR1xCD**	0.08676	1.83561	0.06456	0.05578	0.83540	0.38353	0.74417	20.78618	0.10782
**1**	**39**	**mostra46R-1xCD**	0.12592	1.16645	0.05510	0.15149	1.21462	0.32784	0.95352	0.49524	0.11387
**1**	**41**	**mostraCln1-1xCD**	0.13785	0.49672	0.02616	0.15018	0.70146	0.18823	0.15693	0.95800	0.06617
**1**	**43**	**mostraCln2-1xCD**	0.40320	0.35656	0.03905	0.12331	0.78060	0.18213	0.18479	0.98315	0.07305
**1**	**45**	**mostrad28NR-1xCD**	0.11432	0.72909	0.08586	0.25356	1.01599	0.41762	0.07983	0.40296	0.03740
**1**	**47**	**mostraHDn1HDNR-1xCD**	0.30376	1.19408	0.07047	0.42652	1.37416	0.26429	0.45154	0.40673	0.05959
**1**	**49**	**mostraHDn1TZNR-1xCD**	0.20196	0.66383	0.04024	0.16815	1.09218	0.24614	0.46499	0.28840	0.04440
**1**	**53**	**mostraCCM-NR-1xCD**	0.14178	0.54809	0.05897	0.08598	1.55052	0.30929	0.82986	0.55619	0.12248
**1**	**55**	**mostraHDn2HDNR-1xCD**	0.23187	0.55663	0.10797	0.29985	1.21401	0.48678	0.91093	1.01363	0.06859
**1**	**56**	**mostraHDn2TZNR-1xCD**	0.72818	0.47670	0.15019	0.17973	3.19026	0.52655	1.30601	0.38977	0.16907
