# Supplementary material for: Investigation of Volatiles in Cork Samples Using Chromatographic Data and the Superposing Significant Interaction Rules (SSIR) Chemometric Tool
Source: Biomolecules. 2020 Jun 11;10(6):896. doi: 10.3390/biom10060896 (PMC7355702; doi:10.3390/biom10060896)
Supplement: Supplementary file 1 [file biomolecules-10-00896-s001.zip › biomolecules-788457-supplementary new/Table_S3.pdf]

Table S3. List of samples and the condition of being treated (1) or non-treated (2).
Peak intensities collected for each sample at the 13 variables (Table 2) selected by SSIR procedure.
The PCA of this matrix conducted to Figure 3

			VARIABLES (those selected by SSIR and listed in Table 2 in the article)												
**Treated?**	**Sample #**	**Sample Name**	**1**	**2**	**3**	**4**	**5**	**6**	**7**	**8**	**9**	**10**	**11**	**12**	**13**
**0**	**1**	**mostra1**	282761	27825	107916	100805	150130	169717	112465	384456	107368	77737	75338	656060	797619
**0**	**2**	**mostra2**	267241	16078	132987	97932	153002	161529	105960	400174	117438	83260	70084	597361	711735
**0**	**3**	**mostra3**	244674	3329	89818	59055	121685	112781	81899	353461	111414	60734	51276	699808	730133
**0**	**4**	**mostra4**	156356	3722	64579	49074	86849	71972	71963	255841	98669	57188	32498	901641	915849
**0**	**5**	**mostra5**	236643	5015	71887	59706	113884	112893	74323	333973	79736	58648	51832	786274	782036
**0**	**6**	**mostra6**	484259	3512	207392	184314	194728	279007	66304	544537	175593	114139	119254	996394	898664
**0**	**7**	**mostra7**	252712	4027	110312	87958	112604	109890	86118	347049	141620	76535	47948	713432	729052
**0**	**8**	**mostra8**	305180	17950	149776	141102	151407	213871	76698	387314	171041	89396	89088	503038	491692
**0**	**9**	**mostra9abans**	128002	108103	108682	115826	85647	98945	123030	167298	121609	66599	56369	397112	483718
**0**	**10**	**mostra10abans**	85573	51738	64967	67909	74598	88483	89734	139531	124856	42839	45897	528207	654045
**0**	**11**	**mostra11abans**	132198	120630	89700	128996	103675	116518	139754	180244	112364	49193	63787	430501	528316
**0**	**12**	**mostra12abans**	59693	9494	47663	119385	56578	53624	58852	120221	102110	39130	33415	522375	587333
**0**	**31**	**mostra121NRNOCD**	175770	304795	49288	47667	276730	62419	731655	372207	50239	48400	33344	19002	25510
**0**	**32**	**mostra121RNOCD**	248643	516026	72957	62733	290629	100598	891387	557651	54885	62659	50560	31624	73126
**0**	**34**	**mostra129NRNOCD**	445614	537867	70533	72905	342718	161691	647736	626585	94909	96090	71774	24077	39134
**0**	**35**	**mostra90NRNOCD**	303581	436808	88407	85310	439506	115943	789168	894085	89061	91848	50547	22355	47010
**0**	**36**	**mostra90RNOCD**	557662	909713	128136	123152	493846	208386	1209322	776010	128834	133216	94151	22948	39230
**0**	**37**	**mostra99NRNOCD**	217876	94904	33894	32071	341974	96308	457782	500715	64845	66491	50827	37989	55935
**0**	**38**	**mostra99RNOCD**	314817	963943	114137	106716	385891	111945	984690	525921	68030	69678	52053	23823	32109
**0**	**40**	**mostra46R-NOCD**	326817	207362	61750	61171	749942	113550	881917	1111337	75469	71951	50503	25314	49502
**0**	**42**	**mostraCln1NR-NOCD**	58636	40371	23313	19781	666869	41413	910299	390880	34330	34607	21424	30317	71549
**0**	**44**	**mostraCln2NR-NOCD**	273012	94475	29562	27032	717913	139984	966441	472957	74548	74629	58764	31154	58685
**0**	**46**	**mostrad28NR-NOCD**	383105	316584	75565	72008	367289	132325	652761	708814	115434	111701	67614	31510	51074
**0**	**48**	**mostraHDn1HDNR-NOCD**	365625	576233	75590	67196	419627	138398	1088992	564918	97356	97905	65443	29737	61744
**0**	**50**	**mostraHDn1TZNR-NOCD**	159487	300440	40961	40206	320805	76354	621954	437340	41926	43007	32513	29313	39744
**0**	**51**	**mostraHDn2HDNR-NOCD**	254538	88848	42678	39475	318301	99834	1168968	459878	96720	98324	54221	34414	62242
**0**	**52**	**mostraHDn2TZNR-NOCD**	387785	101223	45990	46568	354150	141194	810981	540290	113014	119046	68223	30254	55956
**0**	**54**	**mostraCCM-NR-NOCD**	350332	87854	36828	31110	152133	112747	346606	573775	103091	105641	52894	36972	53184
**1**	**13**	**mostra1desp**	1830487	2227804	1080470	1036903	1058752	1886769	2034049	1743283	370455	322757	752485	380548	360411
**1**	**14**	**mostra2desp**	1718749	2596000	1075832	996467	1031285	1880699	2419732	1604407	351487	315635	746143	199367	210501
**1**	**15**	**mostra3desp**	2051783	2446713	1094620	1054801	1138935	2071230	2264314	1970506	354948	322717	842916	397010	357943
**1**	**16**	**mostra4desp**	1928216	1568957	1026886	993673	1046240	1916569	1356214	1845514	370819	324548	791689	232233	283202
**1**	**17**	**mostra5desp**	1942142	1300501	877617	847641	1013575	1797328	1188052	1816075	344603	301959	696467	362814	426233
**1**	**18**	**mostra6desp**	760010	1.2E+07	900083	966633	526453	632544	7225120	566343	265538	211351	380755	368237	507671
**1**	**19**	**mostra7desp**	1030485	1.7E+07	1535715	1719024	839077	919624	1E+07	727132	410400	323343	605359	245649	302697
**1**	**20**	**mostra8desp**	1042508	1.3E+07	1316726	1478726	943003	983714	8077439	726391	375925	302970	664662	162571	228545
**1**	**21**	**mostra9desp**	763593	5995299	570104	707550	587380	660553	3801051	564804	209296	161626	466018	410611	412074
**1**	**22**	**mostra10desp**	975793	8502012	892562	1109612	800276	889211	5307597	704249	288323	220467	615135	345759	377548
**1**	**23**	**mostra11desp**	769091	8043047	739372	839659	611792	698559	5161735	568778	245882	189342	454968	309761	368433
**1**	**24**	**mostra12desp**	209786	9386040	623004	704481	269520	143519	6460804	398822	129546	107244	85335	244862	239854
**1**	**25**	**mostra121NR**	892567	483958	202662	196398	573044	289909	1886067	973225	378819	378271	135341	32323	50096
**1**	**26**	**mostra121R**	975283	612721	215535	211337	594322	352043	1449482	1005940	415846	413988	147429	25323	33265
**1**	**27**	**mostra90NR**	1034690	2478837	571429	571445	524254	374108	2246581	1109371	472354	476772	165993	29475	36402
**1**	**28**	**mostra90R**	975474	1126194	432730	421324	529902	393522	1918034	1117023	486466	509984	175185	34806	41778
**1**	**29**	**mostra99R**	1221289	2494134	522406	517392	472452	328475	2305242	1233927	532869	531311	158663	25985	35459
**1**	**30**	**mostra99NR**	1803340	2738087	888168	893380	918870	658155	2575790	1924879	840602	836216	294077	28798	32730
**1**	**33**	**mostra129NR1xCD**	1079549	3449410	579910	578132	372513	333918	3152968	997207	444203	444309	155916	25283	29687
**1**	**39**	**mostra46R-1xCD**	1891319	4702483	739936	734679	580241	561265	4115782	1722662	705871	708591	243751	26373	36158
**1**	**41**	**mostraCln1-1xCD**	1245107	2983441	576019	564221	574542	365049	2757619	1373097	471678	482523	173233	21072	29833
**1**	**43**	**mostraCln2-1xCD**	1567464	2537303	628374	612079	600537	658726	2603503	1425137	682127	696602	292493	31758	47559
**1**	**45**	**mostrad28NR-1xCD**	1545221	2607422	594861	597901	555878	526339	2258422	1750112	642844	661231	222370	28046	35819
**1**	**47**	**mostraHDn1HDNR-1xCD**	1415106	3120893	553254	561056	505513	434241	2917474	1544540	525800	522786	195821	25768	30914
**1**	**49**	**mostraHDn1TZNR-1xCD**	1306366	1251769	387254	375099	779201	502019	1470483	1313331	638793	652035	227648	32997	54876
**1**	**53**	**mostraCCM-NR-1xCD**	920056	3805946	584254	566709	382188	313699	3513864	891287	374033	369332	144044	19490	25839
**1**	**55**	**mostraHDn2HDNR-1xCD**	949547	3583815	617167	614489	779487	297333	3520196	1241516	393377	395627	143073	20544	50596
**1**	**56**	**mostraHDn2TZNR-1xCD**	1199301	996768	287798	291279	678273	426082	1389284	1465372	561674	567370	192916	35493	56099
